# Clinical Effect on Cortical Reactivity of Theta Burst Versus High‐Frequency Repetitive Transcranial Magnetic Stimulation in Parkinson's Disease: A TMS‐EEG Study

**DOI:** 10.1111/cns.70605

**Published:** 2025-09-11

**Authors:** Jiajing Wu, Sheng Zhuang, Yinlian Han, Fan Gao, Chengjie Mao, Jing Chen, Chun‐feng Liu

**Affiliations:** ^1^ Department of Neurology and Suzhou Clinical Research Center of Neurological Disease The Second Affiliated Hospital of Soochow University Suzhou China

**Keywords:** EEG, iTBS, PD, rTMS, TMS‐EEG

## Abstract

**Background:**

Both high‐frequency repeated transcranial magnetic stimulation (rTMS) and intermittent theta burst stimulation (iTBS) are believed to enhance cortical excitability. However, clinical comparison of their benefits for motor improvement in Parkinson's disease (PD) as well as their underlying neurophysiological changes remain unelucidated.

**Objective:**

To compare the clinical effect of rTMS versus iTBS on motor improvement and explore their influence on cortical reactivity using resting state electroencephalogram (EEG) and TMS‐EEG.

**Methods:**

Idiopathic PD patients were randomized in a single‐blind, controlled trial, and received 10 sessions of either 10‐Hz rTMS or iTBS applied to the bilateral primary motor cortex (M1). Motor symptoms were evaluated using the Unified Parkinson's Disease Rating Scale. Cortical reactivity was assessed by resting state EEG and TMS‐evoked potentials.

**Results:**

Included were 52 PD patients (mean [SD] age 66.2 [5.8] years). Compared to 10 Hz rTMS, iTBS was more efficient in alleviating tremor symptoms. In the iTBS group, the global mean field amplitude of P60 was significantly higher after stimulation over contralateral M1 of the affected side and was inversely correlated with motor improvement. In the 10 Hz rTMS group, the global mean field amplitude of P30 evoked by ipsilateral M1 increased significantly. The combination of oscillation and connectivity of *δ* frequency in the frontal cortex of PD provided a decent model in predicting iTBS responders at baseline.

**Conclusion:**

Compared with high‐frequency rTMS, iTBS applied to M1 was more effective in improving tremor symptoms in PD. The two non‐invasive stimulation modalities appear to exert their effects through distinct neurophysiological pathways, as measured by EEG and TMS‐EEG.

## Graphical abstract

iTBS and high‐frequency rTMS may benefit motor symptoms of PD through different neurophysiological effects measured by TMS‐EEG.

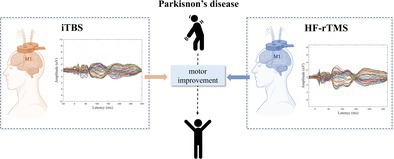

## 1 Introduction

Parkinson's disease (PD) is the second most common neurodegenerative disorder primarily affecting motor function including tremor, bradykinesia, rigidity, and postural instability [1]. Currently, treatment for PD primarily relies on medication but is limited for its potential side effects such as levodopa‐induced dyskinesia, symptom fluctuation, or psychiatric issue [2]. In this context, transcranial magnetic stimulation (TMS) is emerging as a promising non‐invasive neuromodulation technique for normalizing neural circuits and was found to induce dopamine‐related neuroprotective effects and restore synaptic plasticity in animal models [3, 4]. Clinically, repetitive TMS (rTMS), with either low‐frequency (≤ 1 Hz) or high‐frequency (> 5 Hz) modalities, is considered to decrease or increase cortical excitability, respectively [5, 6]. For PD patients, high‐frequency rTMS over the bilateral primary motor cortex (M1) has been recommended in improving general motor symptoms [7]. However, the potential therapeutic mechanism by which rTMS modulates cortical excitability and synaptic restoration within the motor circuits of PD remains unclear.

In the hope of achieving long‐lasting and immediate benefits from stimulation, efforts have been made to optimize the efficacy of rTMS in PD treatment including applying an accelerated protocol [7]. Theta burst stimulation (TBS) is an intensified patterned burst stimulation where 50 Hz of stimulation is delivered in groups of three consecutive pulses, five times per second. Intermittent TBS (iTBS) can enhance cortical excitability and exhibits even more significant neuro‐modulatory effects than of rTMS in healthy participant [8]. In the aspects of depression alleviation [9, 10], pain relieving [11] and decision‐making capacity [12]. TBS was shown to be not inferior to conventional rTMS and was more clinically effective and less time‐consuming [7, 13]. Nevertheless, there is still a lack of head‐to‐head comparative studies on the effect of traditional high‐frequency TMS and iTBS in PD patients. Whether the two TMS modalities contributed to equivalent or different motor improvement was seldom investigated previously.

Resting state electroencephalogram (EEG) can help to observe the characteristics of changes in cortical excitability in different brain regions with high temporal resolution, which is helpful to explore the neuroelectrophysiological mechanisms from TMS treatment. Deterioration of the nigrostriatal dopaminergic system in PD may lead to the disruption of the cortico‐basal ganglia‐thalamo‐cortical loop. Thus, the EEG‐recorded cortical oscillations can reflect alterations in the above loop even in early PD stages where anatomical changes are not yet evident. Meanwhile, quantitative EEG analysis can provide an objective indicator of cortical activity characteristics in PD as well as biomarkers for evaluating different symptomology [14] and disease progression [15]. In addition, TMS combined with EEG can explore brain states and dynamics in motor and non‐motor cortical areas including cortico‐cortical interactions on a millisecond timescale and interactions between excitatory and inhibitory mechanisms [16]. TMS evoked potential (TEPs) from M1, one of the most extensively studied TMS‐EEG parameters, also contributed to the understanding of motor cortex abnormalities and clinical intervention mechanism in PD [17, 18]. Although age [19] and gender may influence rTMS treatment outcome, the brain state at a personal level, as evaluated by functional connectivity of resting state EEG, is considered a more powerful indicator in predicting the efficacy of rTMS treatment [20, 21]. Therefore, the combination of resting state EEG signal and TEPs to reflect neurophysiological alterations between rTMS and iTBS remains to be explored. The predictive value of baseline EEG biomarkers in TMS treatment efficacy also needs further investigation.

To address the aforementioned knowledge gaps, we thus conducted a randomized controlled trial to compare the clinical effect on motor improvement between iTBS and 10 Hz rTMS on PD patients during a 10‐day intervention. We also applied resting state EEG and TMS‐EEG to assess the immediate (after 1 session) as well as post‐treatment (after 10 sessions) electrophysiological changes on the two modes of stimulation, and explored their correlations with clinical improvement and predictive significance.

## 2 Methods

### 2.1 Participants

A total of 94 eligible PD patients were initially screened from April 2022 to February 2024 from the outpatient in the Second Affiliated Hospital of Soochow University. Patients who met the following inclusion criteria were enrolled: (1) Clinically diagnosed with idiopathic PD according to the 2015 Movement Disorder Society diagnostic criteria [22], H‐Y stage: 2–3; (2) age > 50 years; (3) being able to cooperate with the assessment and treatment; (4) Mini‐Mental State Examination score ≥ 24 (≥ 17 for illiterate patients); (5) with stable use of anti‐Parkinson's medication and other drugs for at least 4 weeks; (6) without history of other severe comorbidities or conditions. Finally, 70 eligible PD patients were enrolled in the current study after screening.

### 2.2 Study Design and Randomization

Our study is an open‐labeled, single‐blind, randomized controlled clinical trial. Randomization was performed using a computer‐generated sequence administered by personnel independent of the study. Participants were randomly assigned to a 1:1 ratio to receive either bilateral M1 iTBS or bilateral 10 Hz rTMS treatment. Only the raters, but not TMS operators or patients themselves, were blinded to treatment allocation during the whole treatment and follow‐ups. The sample size was calculated a priori based on the primary outcome of mean Unified Parkinson's Disease Rating Scale part III (UPDRS‐III) total score change post TMS [23]. Assuming a 5% significance level (two‐sided) and 80% power, a total of 50 participants in each group would be required to detect a clinically relevant difference between the two groups, using an independent *t*‐test (assuming a SD of 2.5). Considering an anticipated 10% dropout rate, the final targeted total sample size was set at 55 participants.

### 2.3 Ethnical Statement

This study was approved by the Ethics Committee of the second affiliated hospital of Soochow University (JK‐LK‐2020‐079‐01) and has been formally registered (ChiCTR2000039557). Written informed consent was obtained by patients themselves or their caregivers prior to their inclusion in the study.

### 2.4 Clinical Assessment

Demographic information including age, gender, and education level was collected at baseline. Disease duration and levodopa equivalent dose were retrieved from recent medical records. Body weight and height were examined at the scene. Motor symptoms were evaluated using the UPDRS‐III and Timed Up and Go (TUG) tests. Subitems of motor symptoms were also recorded: upper limb score (UPDRS‐III item 20–25), lower limb score (UPDRS‐III item 20, 22, 26, 29), axial score (UPDRS‐III item 27–30), tremor (UPDRS‐III item 20, 21), rigidity (UPDRS‐III item 22), postural instability (UPDRS‐III item 30), akinesia (UPDRS‐III item 23–26), bradykinesia (UPDRS‐III item 31). All assessments were conducted at a fixed time during the “ON” state of the PD patients. Clinical assessments were performed at baseline, after 10‐session treatment, and 1 month after the treatment by trained doctors blinded to the study.

### 2.5 rTMS and iTBS Procedure

Magstim Rapid stimulator (Magstim, UK) with a figure‐eight coil (MCF‐B70) was applied in our study. Resting motor threshold (RMT) on bilateral primary motor cortex was measured. RMT was determined as the minimum visible contraction of the abductor pollicis brevis muscle. The coil handle was angled at 45° to the midline, pointing backward. iTBS was set at 80% RMT, with each pulse containing 3 stimuli at 50 Hz. Each side received 20 trains with 8‐s intervals, totaling 1200 stimuli over 6 min and 18 s. Routine rTMS treatment involved bilateral M1 area stimulation at 10 Hz, with an intensity of 90% RMT. On each side, patients received 20 trains of 30 stimuli with a 40‐s interval between sequences, totaling 1200 pulses over 27 min and 16 s. In both groups, patients had 10 sessions of treatments. During TMS treatment, any adverse event will be recorded based on the updated 2020 expert guidelines on the safety and recommendation [24] including seizures, headaches, hearing loss, tinnitus, and dizziness.

### 2.6 EEG Recording and TMS‐EEG

EEG recording was conducted in a quiet room. An EEG cap with 59 ring electrodes (Neuracle, China) was used to record the EEG data. Patients were asked to sit in a comfortable chair with muscles relaxed and earplugs worn and remain awake with their eyes closed for a 7‐min resting state EEG recording. The sampling frequency was set at 1000 Hz, resistance below 5000 Ω. TEPs were recorded with single‐pulse TMS combined with synchronized EEG. A 70 mm figure‐eight coil (MCF‐B70) was used to deliver each TMS pulse. Each TMS‐EEG set comprised 100 pulses with an average 3 s intervals. Stimulation targeted bilateral M1, with the coil at a 45° angle to the scalp. The contralateral M1 of the more affected upper limb was defined as M1(+) while the ipsilateral M1 as M1(−). Stimulation intensity was set at 90% of the motor evoked potential. A 0.5 cm foam layer under the coil was used to minimize bone‐conduction noise and scalp vibration sensations. EEG data were recorded three times at baseline, 5 min after the first session, and the completion of 10 sessions of treatment (Figure 2).

### 2.7 EEG Data Analysis

Preprocessing of resting state EEG data followed the recommended procedures of EEGLAB v2023.1. EEG oscillatory activities were classified by frequency bands: *δ* (1–4 Hz), *δ*1 (1–2 Hz), *δ*2 (2–4 Hz), *θ* (4–8 Hz), *α* (8–13 Hz), *β* (13–32 Hz), and *γ* (32–45 Hz). The metric to assess EEG activity slowing was calculated: ratio 1 = (*δ* + *θ*)/(*α* + *β*) [25]. Power spectral density (PSD) for resting‐state EEG signals across frequency bands was calculated using the pwelch function. A sampling frequency of 1000 Hz was applied to two‐second segments. Relative PSD was determined by dividing the absolute PSD of a specific frequency band by the total PSD. Phase Lag Index (PLI), used as a parameter for measuring functional connectivity, is calculated based on the asymmetry of the instantaneous phase difference distribution between two brain regions [26].

TMS‐EEG signals were preprocessed using EEGLAB v2023.1 and the open‐source toolbox TESA v1.1.1 [27]. Data were segmented into epochs from −1000 to 1000 ms around the TMS pulse. To remove artifacts from the magnetic field, interpolation was applied to data from −5 to 15 ms. Data underwent manual inspection and two rounds of Independent Component Analysis (ICA) using FastICA. A semi‐automatic component selection algorithm (tesa_compselec) was used to remove artifacts caused by muscle activity, eye movement, and electrical noise. The TEPs from all electrodes were averaged to obtain the global mean field amplitude (GMFA). The calculation formula is as follows (*K* representing the total number of electrodes) [28].

$$\text{GMFA}\left(t\right)=\sqrt{\sum_{i}^{K}\frac{{\left(V_{i}\left(t\right)-V_{\text{mean}}\left(t\right)\right)}^{2}}{K}}$$

Local TEPs were calculated by averaging signals from electrodes nearest to the stimulation site: M1 left (FC5, FC3, FC1, C5, C3, C1, CP5, CP3, CP1) and M1 right (FC6, FC4, FC2, C6, C4, C2, CP6, CP4, CP2) [29]. Peak values were defined based on previous literature [30].

### 2.8 Outcome Measurement

The primary outcome of our study is the change of UPDRS‐III score and alteration of resting state EEG signals and TEPs after 10‐session TMS treatment. The secondary outcome is the change of UPDRS‐III total score and its subscore 1 month after TMS intervention and immediate changes of resting‐state EEG signals and TEPs 5 min after the first TMS session.

### 2.9 Statistical Analysis

Statistical analysis was performed using IBM SPSS Statistics (Version 27.0.1, USA). Statistically significant difference was defined as two‐tailed *p* < 0.05. The Shapiro–Wilk test was used to test data normality. Normally distributed data were presented as mean ± standard deviation, whereas non‐normal data were presented as median (interquartile range). Independent *t*‐test or Mann–Whitney *U* test was used for normally and non‐normally distributed data, respectively. Categorical variables were presented as frequency (percentage) and were analyzed using chi‐square test. For continuous data measured at multiple time points, generalized estimating equations were used for non‐normally distributed data. Group by time interactions were analyzed via simple effect analysis. When interaction effects were absent, main effects were analyzed. Within‐group differences were analyzed using paired *t*‐tests with Bonferroni correction. Pearson correlation or Spearman correlation analysis was used to explore the relationship between clinical scale scores and resting‐state EEG and TEPs after 10 sessions.

Given the interindividual TMS response variability, we further classified PD patients into responders and non‐responders based on the change of UPDRS‐III scores after 10 sessions. Responders were defined as those with a score improvement rate ≥ 30% or an absolute score change > 5 points in UPDRS‐III [31, 32]. To predict response status, we applied a machine learning model using resting‐state EEG and/or TEPs to classify PD patients as responders or non‐responders after TMS treatment. Specifically, we used the resting‐state EEG coherence matrix as input for the feature‐based machine learning model, with the change of UPDRS‐III scores as the response variable for classification. A linear support vector machine classifier implemented 5‐fold nested cross‐validation (1000 repetitions for result averaging). Model performance was evaluated by balanced accuracy and area under the curve (AUC).

## 3 Results

### 3.1 Baseline Data

**FIGURE 1:**
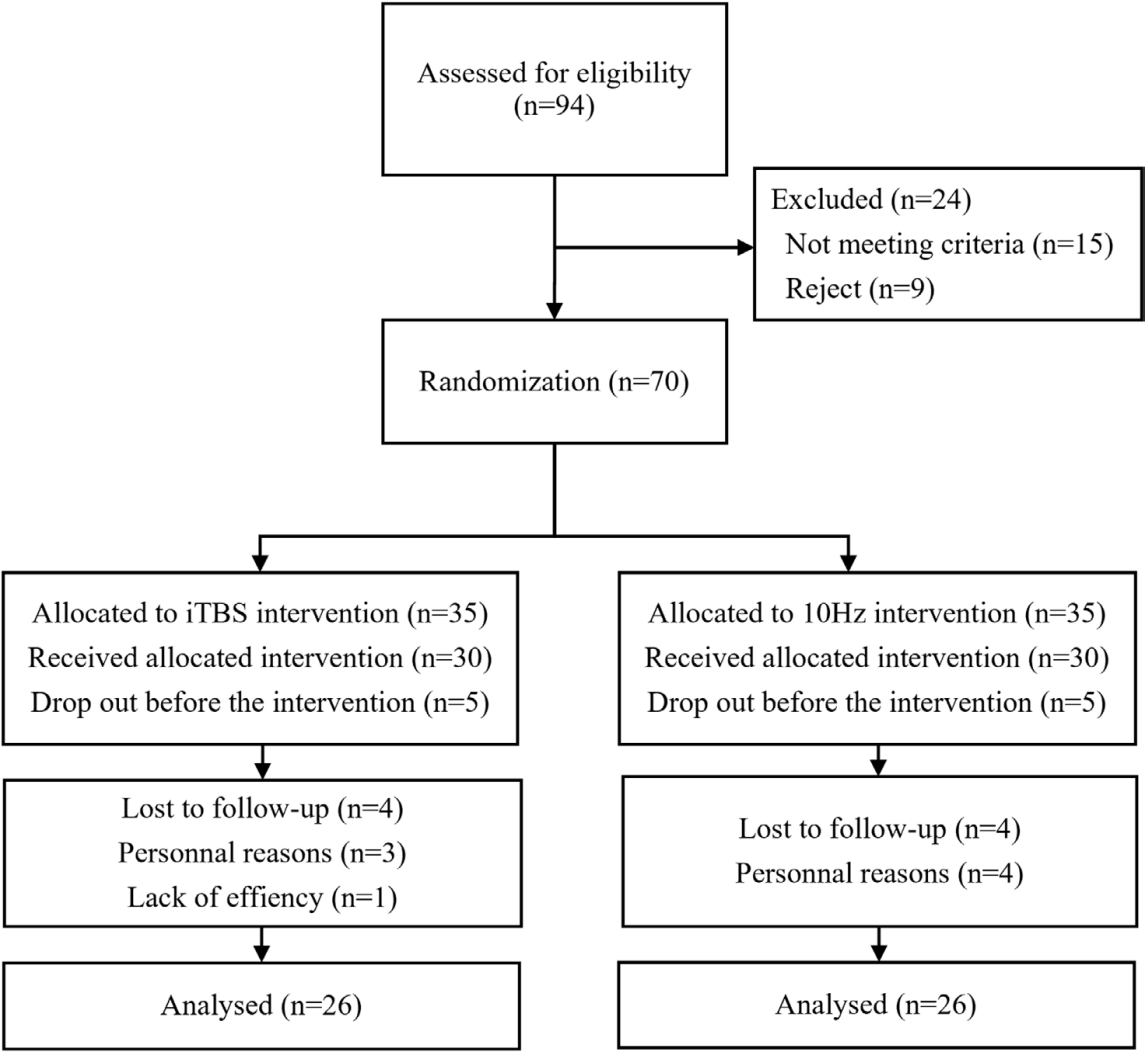
The CONSORT flow diagram of enrolled participants. iTBS, intermittent theta burst stimulation; rTMS, repetitive transcranial magnetic stimulation.

A total of 70 PD patients were randomly assigned into the iTBS group (*n* = 35) or 10 Hz rTMS (*n* = 35) group. Finally, 52 patients completed the treatment and follow‐up and were enrolled in the final analysis (Figure 1). The mean age was 66.2 years, and 30 of them were male. Their average disease duration was 74.0 months, with a mean H‐Y stage of 2 and an UPDRS‐III score of 21.5 points. At baseline, no significant differences were noticed in age, gender, disease duration, levodopa equivalent dose, or H‐Y stage between the two groups (Table 1). Similarly, baseline UPDRS‐III total scores and their subscores did not differ significantly between groups.

**TABLE 1:** Baseline demographic and clinical features of patients.

	iTBS	10 Hz rTMS	*p*
Gender, male (%)	15.0 (57.6)	15.0 (57.6)	0.557
Age (years)	66.5 ± 6.0	66.0 ± 5.7	0.778
Disease duration (months)	91.4 ± 56.2	70.7 ± 35.6	0.119
Education (years)	9.0 (7.8–12.8)	9.0 (4.8–13.0)	0.270
Body Mass Index (kg/m^2^)	23.2 (21.1–26.5)	24.1 (21.2–27.4)	0.504
Levodopa equivalent dose (mg)	550.0 (356.3–781.3)	475.0 (393.8–680.7)	0.763
H‐Y stage	2.0 (2.0–3.0)	2.0 (2.0–2.5)	0.555
UPDRS‐III	19.0 (15.0–29.8)	22.0 (13.8–32.3)	0.798
Upper limbs	9.0 (5.8–12.0)	8.0 (6–14.3)	0.659
Lower limbs	4.0 (3.0–7.3)	6.0 (2.8–8)	0.685
Axial	3.0 (1.0–4.0)	3.0 (1.0–5.0)	0.911
Tremor	2.5 (1.0–6.0)	2.0 (1.0–4.0)	0.161
Rigidity	4.0 (3.0–6.0)	6.0 (3.0–9.0)	0.057
Postural instability	0.0 (0.0–1.0)	0.0 (0.0–1.0)	0.984
Akinesia	5.5 (3.0–11.3)	6.0 (4.0–12.0)	0.550
Bradykinesia	1.0 (1.0–2.0)	1.0 (1.0–2.0)	0.854
TUG (s)	11.2 (9.7–12.9)	11.5 (9.9–12.6)	0.949

Abbreviations: H‐Y, Hohen‐Yahr stage; iTBS, intermittent theta burst stimulation; rTMS, repetitive transcranial magnetic stimulation; TUG, Timed Up and Go test; UPDRS‐III, Unified Parkinson Disease Rating Scale part III.

### 3.2 Comparison of the Changes of Motor Symptoms

A significant interaction effect (time × group) was observed on the tremor subscore (Wald = 6.268, *p* = 0.044; Table 2). Specifically, there was a marginal significant difference (*p* = 0.046) in tremor scores between the two groups 10 days post‐treatment. However, such changes were not statistically significant at follow‐up (*p* = 0.063). In the iTBS group, compared to baseline, symptoms of tremor significantly improved at both the post‐treatment (*p* < 0.001; Table 2) and one‐month follow‐up (*p* = 0.001). In the 10 Hz rTMS group, significant changes for tremor were only observed at the 10‐session post‐treatment timepoint (*p* = 0.002; Table 2). As for other motor aspects, a significant time effect was noticed on the scores of UPDRS‐III (Wald = 70.895, *p* < 0.001), upper limbs (Wald = 46.141, *p* < 0.001), lower limbs (Wald = 22.877, *p* < 0.001), axial (Wald = 9.831, *p* = 0.007), rigidity (Wald = 6.647, *p* = 0.039), postural instability (Wald = 6.647, *p* = 0.037), akinesia (21.098, *p* < 0.001), bradykinesia (6.778, *p* = 0.034), and TUG (Wald = 8.449, *p* = 0.015). There were no significant differences in the aforementioned symptoms between the two groups. No adverse events were reported by patients or their caregivers during the intervention and follow‐up.

**TABLE 2:** Change in motor symptom from baseline to 1‐month follow‐up.

	iTBS			10 Hz rTMS		
	T_0_	T_10_	T_1M_	T_0_	T_10_	T_1M_
UPDRS‐III	19.0 (15.0–29.8)	17.5 (10.8–22.5)^a^	18.0 (11.5–25.0)^a^	22.0 (13.8–32.3)	16.5 (12.0–27.5)^a^	17.5 (11.5–30.3)^a^
Upper limbs	9.0 (5.8–12.0)	6.0 (3.8–9.3)^a^	8.0 (4.8–10.3)	8.0 (6–14.3)	7.0 (5.0–12.0)^a^	8.5 (4.0–13.3)^a^
Lower limbs	4.0 (3.0–7.3)	4.0 (3.0–5.3)^a^	3.5 (2.0–5.0)^a^	6.0 (2.8–8.0)	4.0 (3.0–6.0)^a^	3.0 (2.0–6.3)^a^
Axial	3.0 (1.0–4.0)	3.0 (1.0–4.0)	2.0 (1.0–3.3)	3.0 (1.0–5.0)	2.0 (1.0–5.0)	1.5 (1.0–4.0)^a^
Tremor	2.5 (1.0–6.0)	1.5 (0.0–3.3)^a^	2.0 (0.8–3.3)^a^, ^b^	2.0 (1.0–4.0)	1.5 (0.0–2.0)^a^, ^b^	2.0 (1.0–3.0)
Rigidity	4.0 (3.0–6.0)	4.0 (2.0–6.0)	4.0 (2.8–6.0)	6.0 (3.0–9.0)	5.0 (3.8–7.3)	5.0 (3.0–8.0)^a^
Postural instability	0.0 (0.0–1.0)	0.0 (0.0–1.0)	0.0 (0.0–1)	0.0 (0.0–1.0)	0.0 (0.0–1.0)	0.0 (0.0–1.0)^a^
Akinesia	5.5 (3.0–11.3)	5.0 (2.0–8.3)^a^	5.5 (3.0–8.3)	6.0 (4.0–12.0)	5.0 (2.8–10.0)^a^	6.0 (2.0–11.0)^a^
Bradykinesia	1.0 (1.0–2.0)	1.0 (1.0–1.3)^a^	1.0 (1.0–2.0)	1.0 (1.0–2.0)	1.0 (1.0–2.0)	1.0 (1.0–2.0)
TUG	11.2 (9.7–12.9)	10.9 (9.2–12.7)^a^	10.2 (8.6–12.2)	11.5 (9.9–12.6)	10.3 (9.2–12.5)	10.7 (9.6–13.1)

Abbreviations: iTBS, intermittent theta burst stimulation; rTMS, repetitive transcranial magnetic stimulation; T_0_, baseline; T_10_, after 10‐session treatment; T_1M_, after 1‐month treatment; TUG, Timed Up and Go test; UPDRS, Unified Parkinson's Disease Rating Scale.

^a^ Significant within‐group difference from baseline (Bonferroni correction was used for multiple comparison).

^b^ Significant from baseline between groups.

### 3.3 Comparison of the Changes of Resting EEG and TEPs

**FIGURE 2:**
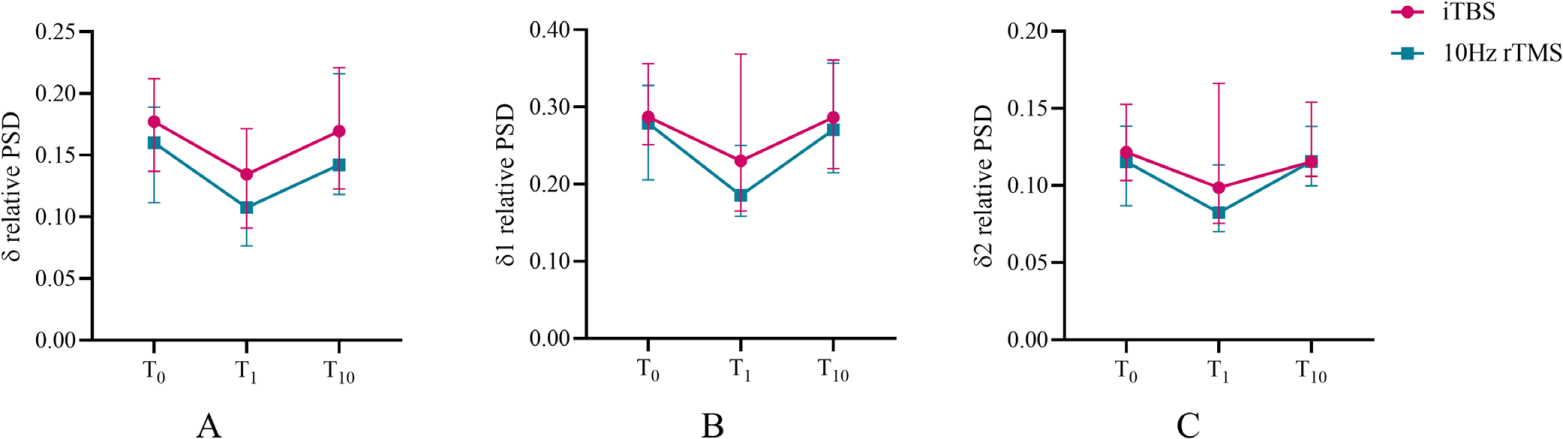
Effects of iTBS and 10 Hz rTMS on resting state oscillation in *δ* frequency after 1‐session (acute) and 10‐session (chronic) stimulation. Generalized estimating equations revealed significant main effect of time on the relative PSD of *δ* (A), *δ*1 (B), *δ*2 (C). In the iTBS group, the relative PSD of *δ* (*p* = 0.004), *δ*1 (*p* = 0.004) decreased after acute stimulation. In the 10 Hz rTMS group, the relative PSD of *δ*2 (*p* = 0.017) decreased after acute stimulation. Both groups did not change significantly after chronic stimulation in the relative PSD of *δ*. iTBS, intermittent theta burst stimulation; PSD, power spectral density; rTMS, repetitive transcranial magnetic stimulation; T_0_, baseline; T_1_, after 1‐session treatment; T_10_, after 10‐session treatment.

As for neurophysiological alterations recorded by EEG, we noticed a significant time effect on *δ* (Wald = 15.194, *p* = 0.001), *δ*1 (Wald = 13.694, *p* = 0.001), *δ*2 (Wald = 13.480, *p* = 0.001), *α* (Wald = 6.467, *p* = 0.039) relative PSD. Multiple comparisons of the main effect of time on PSD across *δ* frequency bands were conducted in both treatment groups at baseline, after the first session, and after 10 sessions of treatment, using Bonferroni correction. In comparison to baseline, *δ* (*p* = 0.004), *δ*1 (*p* = 0.004) relative PSD decreased after the first session in the iTBS group and *δ*2 (*p* = 0.017) relative PSD decreased after the first session in the 10 Hz rTMS group. In both groups, *δ*, *δ*1, *δ*2 relative PSD after 10 sessions of treatment did not differ significantly from the study baseline. The relative PSD in *α* frequency increased significantly after the first session in both groups compared to the baseline (*p* = 0.046) but not after the 10 sessions of treatment (*p* = 0.216; Figure 2).

**FIGURE 3:**
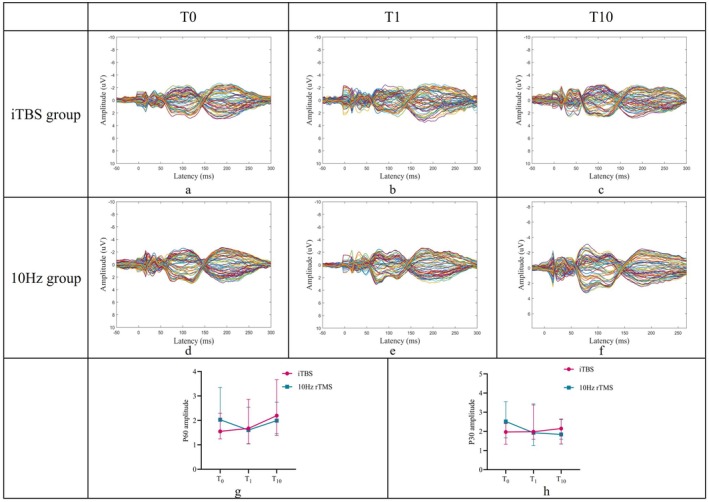
Effects of iTBS and 10 Hz rTMS on TEPs after 1‐session (acute) and 10‐session (chronic) stimulation. (a–c) TEPs from stimulation of the M1 contralateral (M1+) to the most bradykinetic upper limb in the iTBS group. (d–f) TEPs from stimulation of the M1 ipsilateral (M1−) to the most bradykinetic upper limb in the iTBS group. (g) In the iTBS group, P60 (M1+) increased after chronic stimulation when compared within and between groups. (h) In the 10 Hz rTMS group, P30 (M1‐) decreased after chronic stimulation when compared within and between groups. iTBS, intermittent theta burst stimulation; M1, primary motor cortex; rTMS, repetitive transcranial magnetic stimulation; T_0_, baseline; T_1_, after 1‐session treatment; T_10_, after 10‐session treatment; TEP, TMS evoked potential.

With TMS‐EEG, we found an interaction effect on GMFA of P30 (Wald = 7.779, *p* = 0.020), P60 (Wald = 9.307 *p* = 0.010) over M1(+) and P30 (Wald = 11.640, *p* = 0.003) over M1(−). We performed a simple effects analysis on P60 at the M1(+) side. The difference of P60 over M1(+) between 10‐session post‐treatment and baseline was statistically significant between the two groups (iTBS > 10 Hz rTMS, *p* = 0.005). In the iTBS group, GMFA of P60 was significantly increased after 10‐session treatment compared to the baseline (*p* = 0.036). There were no significant changes after rTMS stimulation compared to the baseline in the 10 Hz group. The difference of GMFA of P30 over M1(−) between 10‐session posttreatment and baseline was statistically significant between the two groups (10 Hz rTMS < iTBS, *p* = 0.023). Significant differences were also found when comparing GMFA of P30 over M1(−) at 10‐session treatment with baseline only in the 10 Hz rTMS group (*p* = 0.023) but not in the iTBS group (Figure 3).

### 3.4 Correlation of the Changes Between Clinical Symptoms and EEG Data

**FIGURE 4:**
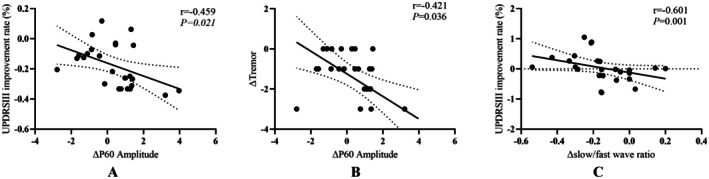
Correlation of EEG changes with symptom improvement after 10‐session treatment for iTBS and 10 Hz rTMS. In the iTBS group, the change in P60 amplitude in the motor area was negatively correlated with the change in UPDRS‐III improvement rate (A) and the change in tremor scores (B). In the 10 Hz rTMS group, the change in slow/fast wave ratio was negatively correlated with the change in UPDRS‐III improvement rate (C). iTBS, intermittent theta burst stimulation; rTMS, repetitive transcranial magnetic stimulation; UPDRS‐III, Unified Parkinson's Disease Rating Scale part III.

In the iTBS group, we found a significant inverse relationship between change in amplitude of P60 over M1(+) in the motor area and change of tremor scores (*r* = −0.421, *p* = 0.036) and UPDRS‐III improvement rate (*r* = −0.459, *p* = 0.021). In the 10 Hz rTMS group, we found a significant inverse relationship between change of ratio 1 and the UPDRS‐III improvement rate (*r* = −0.601, *p* = 0.001; Figure 4).

### 3.5 Difference and Predictive Model Between Responder and Non‐Responder

**FIGURE 5:**
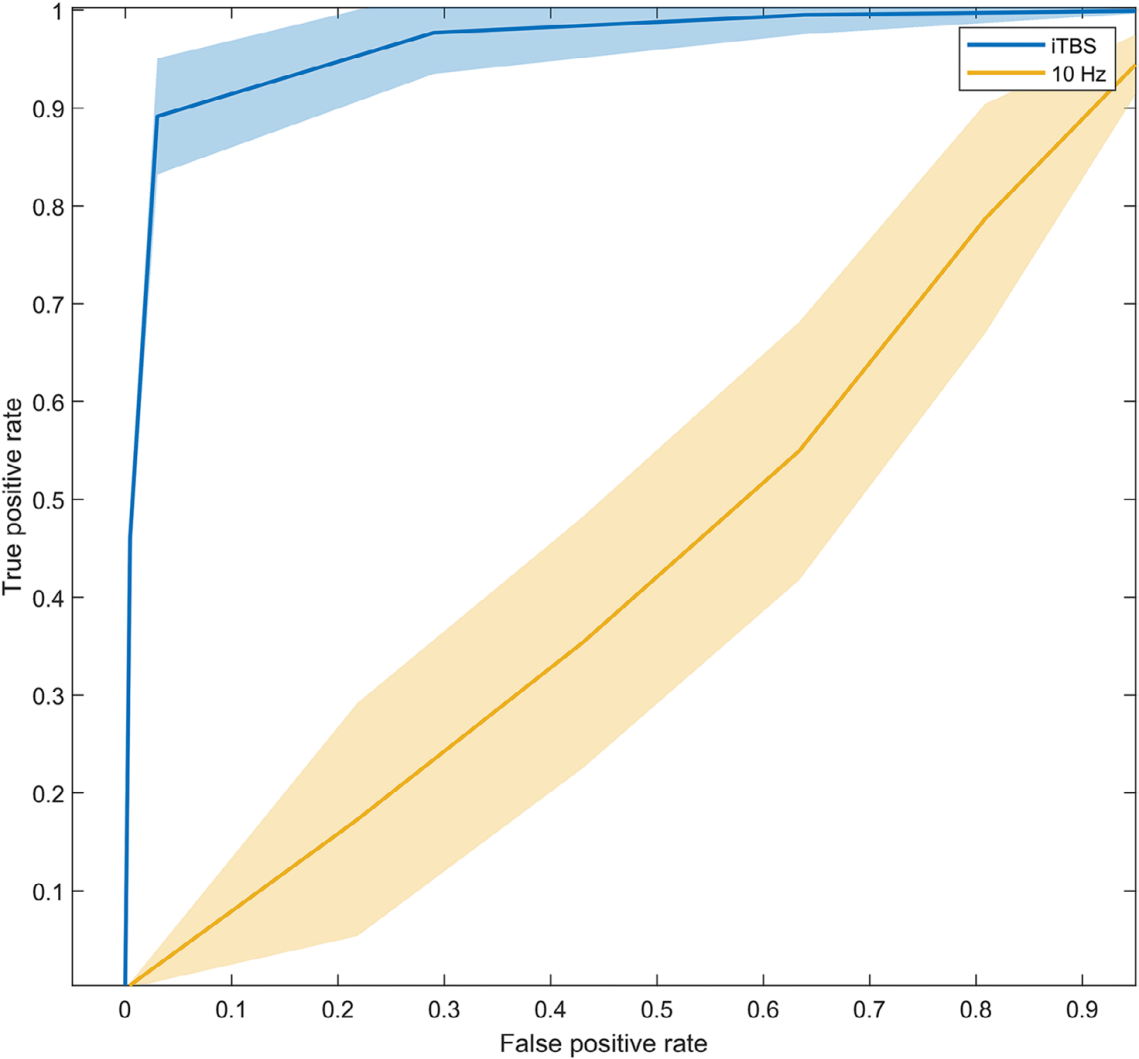
ROC curve of combined characteristic of electroencephalogram to predict responder and non‐responder in the iTBS group. Frontal *δ* relative power spectral density and phase lag index were used to create a model that classified responder and non‐responder after iTBS. The area under the curve was of 0.972, with a sensitivity of 0.901, and specificity of 0.925. iTBS, intermittent theta burst stimulation; ROC, receiver operating characteristic curve; rTMS, repetitive transcranial magnetic stimulation.

A significant difference in baseline relative PSD of the frontal *δ* frequency band (0.355 ± 0.111 vs. 0.442 ± 0.088, *p =* 0.034) was found between responders and non‐responders only in the iTBS group. The responders also had significantly lower PLI values at the F2–F7 *δ* frequency compared to non‐responders (*P*_FDR_ < 0.001). When combining the above features of the oscillation and connectivity of *δ* in the frontal cortex, the performance of the current model was decent in predicting iTBS responders with an estimated accuracy of 0.931 and AUC of 0.972 (Figure 5).

## 4 Discussion

In this study, we found that bilateral M1 targeted iTBS might be better than 10 Hz rTMS in improving tremor but not general motor symptoms in PD, which effect can last for at least 1 month. Using resting state EEG, the time effect of *δ* and *α* relative PSD was noticed on two TMS modalities. Their neurophysiological effect might exert through different pathways as reflected on P60 evoked by M1(+) or P30 evoked by M1(−) based on TMS‐EEG. These alterations were also correlated with clinical motor improvement in PD. Additionally, markers of resting state EEG signals might serve as indicators for screening potential responders of iTBS treatment at baseline.

Previous studies have demonstrated that high‐frequency rTMS applied to bilateral M1 can improve motor symptoms in PD patients [31, 33], which were also consistent with our findings. However, some also indicated limited efficacy of high‐frequency M1‐targeted rTMS for tremor and axial symptoms [34, 35]. In our study, iTBS seemed to be superior in dealing with tremor in patients compared with 10 Hz rTMS. Notably, iTBS enhances cortical excitability during both “ON” and “OFF” stages in PD [36] and offers significant time and cost efficiencies compared to traditional rTMS. Given these advantages, iTBS may represent another clinical option for managing PD tremor. Furthermore, multiple daily iTBS sessions may enhance therapeutic efficacy [13]. Despite relatively better performance on tremor for iTBS over rTMS in the current study, the exact mechanisms remain unclear. As iTBS may work by inducing long‐term potentiation‐like plasticity in the M1, the modality might increase short‐interval intracortical inhibition and thus normalize the cerebello‐thalamo‐cortical connectivity [37, 38], which is correlated to tremor pathogenesis in PD. Therefore, more studies are warranted to determine whether such findings were due to chance or actual neurophysiological links.

In our cohort, both iTBS and 10 Hz stimulation significantly reduced *δ* band relative PSD after the first stimulation session, rather than following the complete 10‐session regimen. Similar acute reductions in *δ* oscillation were observed in healthy participants after iTBS or 10 Hz rTMS [39, 40]. As *δ* oscillation is considered a marker of cortical inhibition leading to suppressed neuronal activity [41], our findings suggest cortical disinhibition after TMS intervention. *δ* oscillations also correlate with dopaminergic depletion in awake mice [42]. Acute dopamine release was observed 5 min post‐continuous TBS in monkeys [43] and post‐acute high‐frequency stimulation, but not chronically [44]. This implies that the acute, non‐linear changes in *δ* relative PSD may relate to transient dopamine release. We speculate that acute spectral oscillation changes may result from dopaminergic release following bilateral M1 stimulation, while chronic stimulation maintains resting‐state cortical activity at a stable physiological set point. The absence of spectral power changes in some studies comparing post‐treatment states to baseline may be explained by this phenomenon. However, we cannot exclude the possibility that resting EEG inadequately reflects TMS‐induced cortical excitability changes, limiting its sensitivity to long‐term effects. Additionally, our small sample size may account for the lack of significant post‐treatment effects.

We also observed an increase in GMFA of P60 evoked by M1(+) in the iTBS treatment group, which was especially significant after the 10‐session treatment. The mechanism of iTBS may involve enhanced activation of specific N‐methyl‐D‐aspartate (NMDA) receptor subunits, which are critical modulators of structural plasticity during synapse development [45, 46]. Pharmacological studies link the M1‐evoked P60 potential to NMDA/glutamate activation [47], and its amplitude is sensitive to cortical excitability changes [48]. In the iTBS group, the progressive increase of P60 amplitude paralleled enhanced cortical excitability. Stimulating M1, the primary input to the corticospinal tract, likely underpinned symptom improvement. Increased P60 amplitude in the motor cortex correlated with improvements in overall motor symptoms and tremor. From this perspective, TMS‐EEG may elucidate potential iTBS mechanisms in PD and provide an electrophysiological marker for monitoring treatment response.

Conversely, in the 10 Hz rTMS group, there was a significant decrease in GMFA of P30 evoked by M1(−), especially significant after 10 sessions of treatment. P30 may be related to GABA (γ‐aminobutyric acid)‐A mediated inhibition and glutamate‐mediated excitation [49]. In vitro, 10 Hz rTMS reduced GABAergic synaptic input strength in CA1 pyramidal neurons by regulating postsynaptic gephyrin scaffolds and dendritic spines [50]. While rTMS is often associated with long‐term potentiation‐like excitability enhancement, our results suggest 10 Hz rTMS may facilitate neurons primarily by reducing cortical inhibition. Furthermore, motor symptom changes negatively correlated with the slow/fast wave ratio in this group. Slowed resting state oscillatory activity is a stable feature of non‐demented PD [51], though its relationship to disease severity remains unclear. The decreased slow/fast wave ratio may also reflect reduced cortical inhibition post‐10 Hz rTMS. The divergent TEPs changes between iTBS and 10 Hz rTMS groups likely reflected distinct neurophysiological responses to each stimulation modality, consistent with initial studies on differing cellular targets [52, 53]. These EEG findings suggest directions for individualized TMS protocols and identify potential biomarkers for closed‐loop therapy.

In the explorative analysis, we also found that, within the iTBS group, responders exhibited lower baseline relative *δ* PSD and frontal *δ* frequency connectivity than non‐responders. A machine learning model then showed promising efficacy in predicting iTBS response, indicating promising phenotypic characteristics in responsive PD patients. Future research could explore interventions to reduce frontal *δ* oscillation and functional connectivity prior to iTBS to potentially enhance efficacy. Nevertheless, models with larger data are needed to validate our findings to give more convincing evidence.

The strengths of the current study include a head‐to‐head comparison of the clinical efficacy of iTBS versus rTMS in PD and the application of EEG and TMS‐EEG to assess differential neurophysiological alterations induced by these two modalities. However, several limitations should be kept in mind when interpreting our results. First, the current study was a single‐centered and single‐blind trial. Therefore, the effect of sham stimulation and the generalizability of our findings are unclear. Second, while resting‐state EEG changes followed a consistent trend, the absence of a sham group also prevented exclusion of potential confounding influences (e.g., environmental comfort) on these oscillatory changes. Third, the limited sample size used in the exploratory machine learning analysis constrains the robustness of the predictive model. Fourth, residual confounding could exist because we did not collect data on certain important factors such as history of depression or sedatives and antidepressants usage, which can change the excitability of M1, thus disturbing the therapeutic effect of TMS. However, all patients in the current study did not change their medications during the study period. This, to some extent, might partially mitigate the influence of medication when interpreting our results.

## 5 Conclusion

In sum, bilateral M1 iTBS may offer advantages over 10 Hz rTMS in improving tremor symptoms in PD patients with sustained efficacy lasting at least 1 month. While both modalities acutely modulated *δ* and *α* relative power spectral density in resting‐state EEG, they exerted distinct neurophysiological effects, as evidenced by differential TMS‐EEG responses. Individualized and quantifiable EEG markers are promising for predicting clinical efficacy in future TMS studies.

## Author Contributions

J.W. and S.Z. conceptualized and designed the study. J.W. and S.Z. drafted the manuscript. Y.H., F.G., and C.M. collected and analyzed data. J.W., S.Z., Y.H., F.G., C.M., J.C., and C.‐f.L. interpreted the data and critically revised the manuscript for the intellectual content.

## Disclosure

The authors have nothing to report.

## Conflicts of Interest

The authors declare no conflicts of interest.

## Data Availability

The data that support the findings of this study are available on request from the corresponding author. The data are not publicly available due to privacy or ethical restrictions.
